# Safety and Early Outcomes of Cochlear Implantation of Nucleus Devices in Infants: A Multi-Centre Study

**DOI:** 10.1177/23312165241261480

**Published:** 2024-06-18

**Authors:** Tal Honigman, Sharon L. Cushing, Blake C. Papsin, Susan Waltzman, Jennifer Woodard, Sara Neumann, Matthew B. Fitzgerald, Karen A. Gordon

**Affiliations:** 1 Department of Otolaryngology, 7979 The Hospital for Sick Children, Toronto, ON, Canada; 2 Department of Otolaryngology-Head & Neck Surgery, 7938 University of Toronto, Toronto, ON, Canada; 3 Department of Otolaryngology-Head and Neck Surgery, New York University Grossman School of Medicine, New York, NY, USA; 4 Department of Otolaryngology/Head and Neck Surgery, University of North Carolina at Chapel Hill, Chapel Hill, NC, USA; 5 Hearts for Hearing, Oklahoma City, OK, USA; 6 Department of Otolaryngology-Head and Neck Surgery, Stanford University, Palo Alto, CA, USA; 7 Department of Communication Disorders, 7979 The Hospital for Sick Children, Toronto, ON, Canada; 8 Program in Neuroscience and Mental Health, 7979 The Hospital for Sick Children, Toronto, ON, Canada

**Keywords:** aided audiogram, IT-MAIS and LittlEARS questionnaires, early treatment, deafness, hearing loss, children

## Abstract

This multi-center study examined the safety and effectiveness of cochlear implantation of children between 9 and 11 months of age. The intended impact was to support practice regarding candidacy assessment and prognostic counseling of pediatric cochlear implant candidates. Data in the clinical chart of children implanted at 9–11 months of age with Cochlear Ltd devices at five cochlear implant centers in the United States and Canada were included in analyses. The study included data from two cohorts implanted with one or two Nucleus devices during the periods of January 1, 2012–December 31, 2017 (Cohort 1, n = 83) or between January 1, 2018 and May 15, 2020 (Cohort 2, n = 50). Major adverse events (requiring another procedure/hospitalization) and minor adverse events (managed with medication alone or underwent an expected course of treatment that did not require surgery or hospitalization) out to 2 years post-implant were monitored and outcomes measured by audiometric thresholds and parent-reports on the IT-MAIS and LittlEARS questionnaires were collected. Results revealed 60 adverse events in 41 children and 227 ears implanted (26%) of which 14 major events occurred in 11 children; all were transitory and resolved. Improved hearing with cochlear implant use was shown in all outcome measures. Findings reveal that the procedure is safe for infants and that they show clear benefits of cochlear implantation including increased audibility and hearing development.

## Introduction

This multi-center study gathered data from children provided with cochlear implants younger than 12 months of age through retrospective chart review with the objective of determining safety and effectiveness of cochlear implantation between 9 and 11 months of age. The intended impact was to support practice regarding candidacy assessment and prognostic counseling of pediatric cochlear implant candidates.

Cochlear implants stimulate the auditory nerve to provide access to sound for individuals who do not benefit from high gain hearing aids often due to severe to profound hearing loss. For children, this hearing enables development of spoken speech and language with outcomes often correlated to the age of implantation due to shorter durations of deafness (Ching et al., 2018; Culbertson et al., 2022; Dettman et al., 2016; Harrison et al., 2005; Kral, 2013; Wu et al., 2023). These data are consistent with the idea that early intervention is crucial for providing access to language for children who are deaf and hard of hearing (Boons et al., 2012; Geers, 2004). Specifically, gaps in language development in children who are deaf and hard of hearing are reduced when the age at identification, diagnosis, and intervention occur within the first 6 months of life (Yoshinaga-Itano et al., 2017, 2018). Similarly, a long-term multi-center study from Australia (LOCHI) confirmed that gaps in outcomes are reduced, although not necessarily eliminated, as age at intervention with hearing aids or cochlear implants decreases (Ching et al., 2018). The need for early intervention is illustrated in the language development of typically developing children, who already respond to spoken words and commonly utter their first expressive word with meaning by 1 year of age (Friedmann & Rusou, 2015). This development requires language exposure and engagement whether it be spoken, manual, or a combination approach (Friedmann & Rusou, 2015; Mayberry, 1993; Romeo et al., 2018).

Development of spoken language specifically requires that children hear language sounds. These are mostly delivered at typical conversational levels and are mostly unavailable for children with severe to profound hearing loss despite high gain hearing aids (Scollie et al., 2005). By contrast, cochlear implants convert the acoustic input into electrical pulses that are delivered within a range of intensities that are audible and comfortable for the user (Rubinstein, 2004). Thus, to provide early intervention for children with severe to profound hearing loss to develop spoken language, the cochlear implant should be provided as soon as possible. Several pediatric cochlear implant centers around the world were already routinely implanting children below the age of 12 months prior to 2020 (Colletti et al., 2011; James & Papsin, 2004; Leigh et al., 2013; Lesinski-Schiedat et al., 2004; Roland et al., 2009) but at that time, guidelines for cochlear implantation in children by the United States Food and Drug Administration (FDA) set the lower limit as 12 months of age. More recently, evidence from the first phase of the present study showed that cochlear implantation in infants aged 9–11 months does not incur increased serious adverse events during the first 6 months post-surgery (Deep et al., 2021; Purcell et al., 2021). These data helped lower FDA recommendations for cochlear implantation using the Cochlear Nucleus device down to 9 months of age in 2020.

In the present study, surgical safety of cochlear implantation at 9–11 months of age was assessed at longer post-operative times and outcomes of cochlear implantation at these young ages were measured. Findings revealed that the procedure is safe in infancy and that these young children show clear benefits of cochlear implantation including increased audibility and hearing development.

## Methods

The study was a retrospective analysis of data in the clinical chart of children implanted at 9–11 months of age with Cochlear Ltd devices at five cochlear implant centers in the United States and Canada (Tables 1 and 2). Each of these investigational sites obtained approval from its institution's designated research ethics board prior to commencing any study-related activities. All sites received training from a Clinical Project Manager at Cochlear Corporation on the protocol, study procedures, data entry system, and good clinical practice. The study included data from two cohorts. Inclusion criteria were: 9–11.99 months of age at the time of cochlear implantation, cochlear implantation between January 1, 2012 and December 31, 2017 (Cohort 1) or between January 1, 2018 and May 15, 2020 (Cohort 2), and cochlear implantation with a Nucleus device. Cohort 1 consisted of 83 children enrolled in the original study (Deep et al., 2021) who had completed the original protocol in which safety outcomes were measured over the first 6 months post-implantation. The present study assessed safety in these same children over a longer period, defined by 24 months after surgery (or date of final protocol approval [May 15, 2020], whichever occurred first) and performance metrics out to the date of final protocol approval (May 15, 2020). Cohort 2 consisted of 50 additional children with more recent implantation who met inclusion criteria. Children implanted outside these time frames or at different ages were excluded from the study.

**Table 1.** Numbers of Children Included in Analyses from Each Cohort and Each Study Site.

Site	Cohort 1	Cohort 2
The Hospital for Sick Children	40	20
New York University Cochlear Implant Center	16	17
University of North Carolina	14	5
Hearts for Hearing	9	7
Stanford University	5	1
Total	84	50

**Table 2.** Demographics of Children in Cohorts 1 and 2.

	Cohort 1 (n = 84)	Cohort 2 (n = 50)
**Age at CI**	n, %	n, %
9 months	28, 33%	20, 40%
10 months	24, 29%	12, 24%
11 months	32, 38%	18, 36%
**Sex**		
Female (n,% of cohort)	42, 50%	21, 42%
**Surgery**		
Simultaneous bilateral CI	61, 73%	32, 64%
Unilateral left CI	12, 14%	6, 12%
Unilateral right CI	11, 13%	12, 24%

Endpoints for this study related to safety and effectiveness of cochlear implantation in children aged 9–11 months at time of implantation. Data were abstracted from medical records and chart notes. All sites used an electronic health record. In some cases, data recorded in paper charts had been scanned and uploaded when the institution implemented an electronic system. All pertinent and available medical records were reviewed. All sites used the same electronic Case Report Form to ensure uniform data collection and reporting. The criteria below were gathered for each subject, and sites indicated if records were not available or if a criterion was “not reported.” Demographic data collected for analyses were: (1) date of birth, (2) date of surgery, (3) sex, and (4) unilateral versus bilateral simultaneous surgery.

Safety of the procedure was collected by assessing surgical details and events (total duration under anesthesia; estimated blood loss; temperature regulation and/or any instances of arrhythmia; facial nerve injury; exposed dura during drilling; skin flap breakdown or extrusion/device migration; device malfunctions; leak of cerebrospinal fluid [CSF]; any other significant complications noted on the operative record), events shortly after surgery (total duration in recovery; amount of pain medication administered via IV in hospital; readmissions to CI center/hospital within 30 days post-surgery for device-or procedure-related complications), and adverse events post-operatively over 2 years (any device-/procedure-/otologic-related untoward medical occurrences including ear-related infections such as acute otitis media). Device deficiencies were noted when any equipment related fault was reported in the electronic health record for 2-years post-operatively.

Effectiveness of cochlear implantation was measured pre-operatively and post-operatively out to 2 years (or the latest visit on file before the 2-year point) by audiometric thresholds and parent questionnaires (LittlEARS and/or IT-MAIS). The LittlEARS is a questionnaire created by MedEl, consisting of 35 yes/no questions (1 point for yes responses, 0 for no) which focus on functional auditory development expected during the first 2 years of life including receptive, expressive, and semantic behaviors (Tsiakpini et al., 2004). The maximum total score is 35. The Infant-Toddler Meaningful Auditory Integration Scale (IT-MAIS) (Zimmerman-Phillips, 2000) consists of an interview with parents/caregivers in response to 10 probing questions which assess vocalization behavior, alerting to sounds, and deriving meaning from sound. Each question has a potential of 0 (lowest) to 4 (highest) points which are based on metrics such as time of demonstrated abilities (e.g., always, more than 50% of the time, less than 50% of the time, or never) which yields a maximum total score of 40. Both measures have high internal consistency and have normative values showing increased scores with age (Coninx et al., 2009; Obrycka et al., 2017; Yang et al., 2020)

### Analyses

Analysis of safety of cochlear implantation was determined by identifying numbers of children who experienced adverse events. Major events were classified as those requiring additional surgery or hospital readmission as compared with minor events that were managed with medication alone or underwent an expected course of treatment that did not require surgery or hospitalization. Events fell into the following categories:

1. Temperature regulation and/or instances of arrhythmia during surgery
2. Post-operative complications
3. Facial nerve injuries
4. Swelling/irritation related to the device(s)
5. CSF leak
6. Device migration/extrusion
7. Readmissions to the hospital
8. Skin flap breakdown
9. Device extrusions
10. Other

Mixed linear models were used to assess effects of cochlear implantation on audibility and parent reported questionnaires of hearing development in R-studio (R Core Team, 2018). Analyses included pre-operative air conduction thresholds from the ear to be implanted at all pure tone frequencies delivered through earphones and tested between 125 and 8000 Hz pre-operatively and aided thresholds at any frequency in the same range with the unilateral or bilateral cochlear implants delivered in the soundfield. To compare thresholds unaided prior to implantation to aided post-operative thresholds, the model included fixed factors of unaided pre-implant versus aided post-implant conditions, test frequency, age at implant and a random intercept per participant was included. Additional effects on unaided thresholds in the ear to be implanted were assessed separately by including fixed factors of investigational site, age at testing, test frequency and test ear, and a random intercept per participant. Effects on aided thresholds were assessed separately for fixed factors of investigational site, test frequency, age at cochlear implantation, and duration of implant use with a random intercept per participant. Differences in questionnaire test scores between pre- and post-implantation per child were assessed for fixed effects of test with random intercept per child and test scores were assessed for fixed effects of duration of implant use, age at CI, questionnaire test type, type of implant (left, right, bilateral), and investigational site with a random intercept per child. Post hoc analyses used the R emmeans library (Lenth, R.V., 2021) which, for factors with more than two levels, employed pairwise t-tests with Kenward–Roger adjustments for degrees of freedom and the Tukey method for p-value adjustments.

## Results

**Figure 1.**
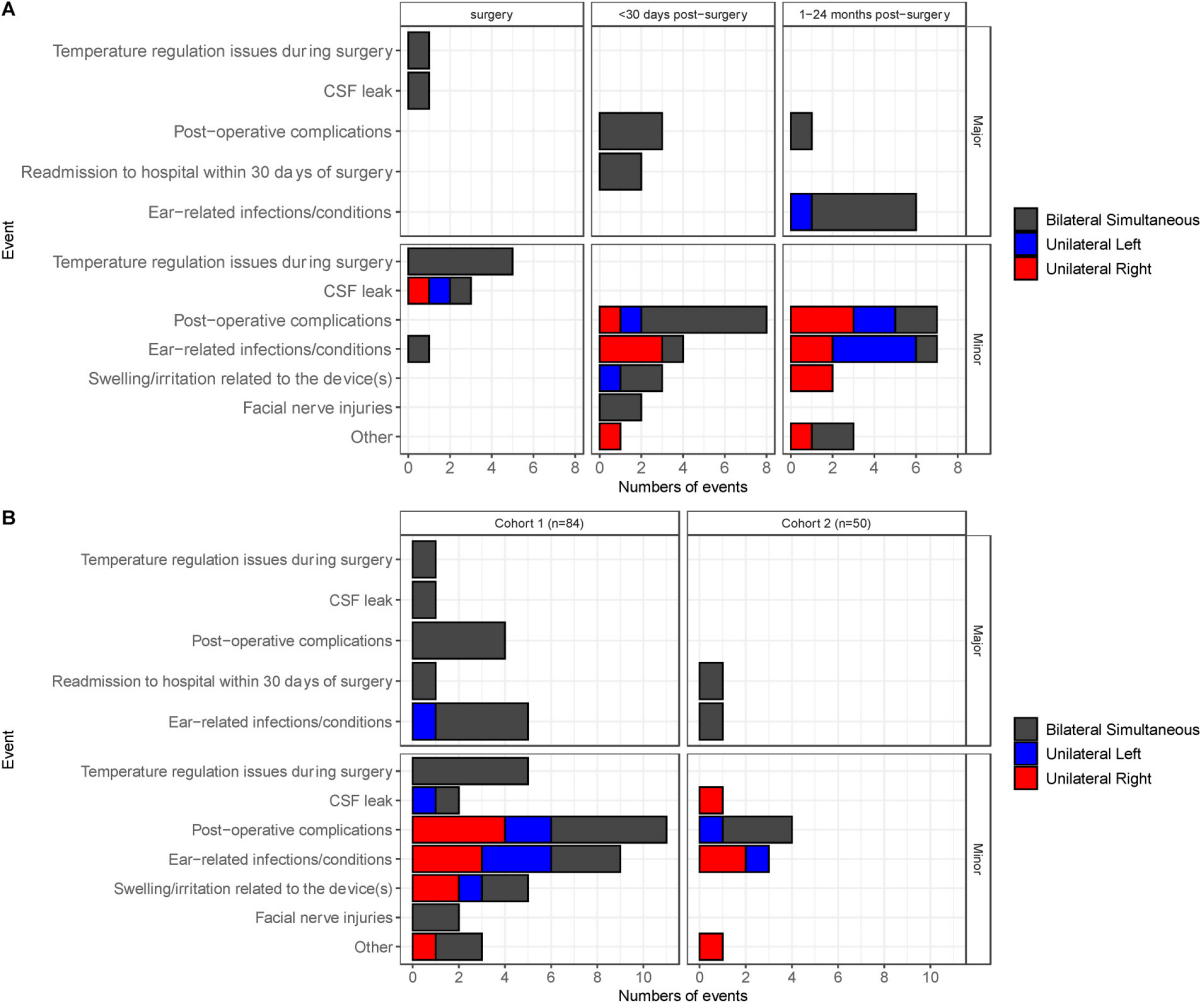
The numbers of major (top row) and minor (bottom row) adverse events for Cohort 1 (left) and Cohort 2 (right) are reported by implantation type. All were transitory and resolved. Post-operative and ear-related infections/conditions were most common.

A total of 134 children with 227 ears implanted were studied. Adverse events were reported in 41 of 134 children (31%). There were a total of 60 adverse events across 227 ears implanted (26%). There were 14 major events (6% of 227 ears implanted) in 11 children; 10 received bilateral CIs in the same surgery and 1 received a unilateral CI for a total of 21 ears implanted. These data have largely been reported (Deep et al., 2021) as nine children were in Cohort 1 and 2 children were in Cohort 2. Events are plotted in Figure 1 by time (A) and Cohort (B). The implant configuration (simultaneous bilateral, unilateral left, or unilateral right) is shown to indicate the numbers of implanted ears. Major and minor events were considered transitory and resolved.

Major events occurred across study sites and at different time points. Most occurred in children receiving bilateral cochlear implants. There was one incident of hyperthermia during the CI surgery that was resolved. One child had a CSF leak that required a return to the operating room the day after CI surgery in one ear (major) and a CSF leak that was managed in the initial surgery in the other ear (minor event reported below). There were three post-operative events in three children (seroma, emesis, fever) that occurred 1 month after surgery and treated in hospital and one post-operative event that required device removal 2 months after seroma. Two children required readmission to hospital in the month following surgery for fever and/or infection at the surgical site for administration of IV antibiotics. At the longer follow-up period (1–24 months post-surgery), there were six ear-related infections (otitis media, mastoiditis, seroma, post-auricular abscess) in four children (seven ears) that were managed by myringotomy and tubes or incision and drainage. Other events (n = 46, 20% of 227 ears implanted) were considered minor.

Minor events in surgery were a need for temperature regulation in five children who were all in Cohort 1. Minor CSF leaks were managed during the initial implant surgery (three events in three children, one of whom had a dysplastic cochlea). Bloody otorrhea was managed during surgery in one ear in one child. Minor post-operative complications (15 events in 12 children) included fever, emesis, and other symptoms of cold or flu, seroma, hematoma, and unsteadiness. None required hospitalization. Minor ear-related post-operative events occurred immediately after surgery to up to 2 years post-implant (12 events in 10 children). These were typically ear infections (acute otitis media) which resolved and did not require hospitalization. Irritation or swelling at the surgical site or skin under and around the transmitting coil device and facial edema (one child) was noted in a total of five events in five children who were all in Cohort 1. Facial nerve weakness was noted in two children in Cohort 1 (one event each) and resolved after treatment with steroids in both cases and antibiotics in addition in one case. Other reported events (four events, three children) were two events of crying/discomfort after CI programming after which C-levels were reduced, one child with a cochlear incomplete partition (Type 1) and cochlear nerve aplasia who received no audibility with the CI, and one child who was sent for genetic testing based on parental reports of unsteadiness and identified with Usher's Syndrome Type 1.

**Figure 2.**
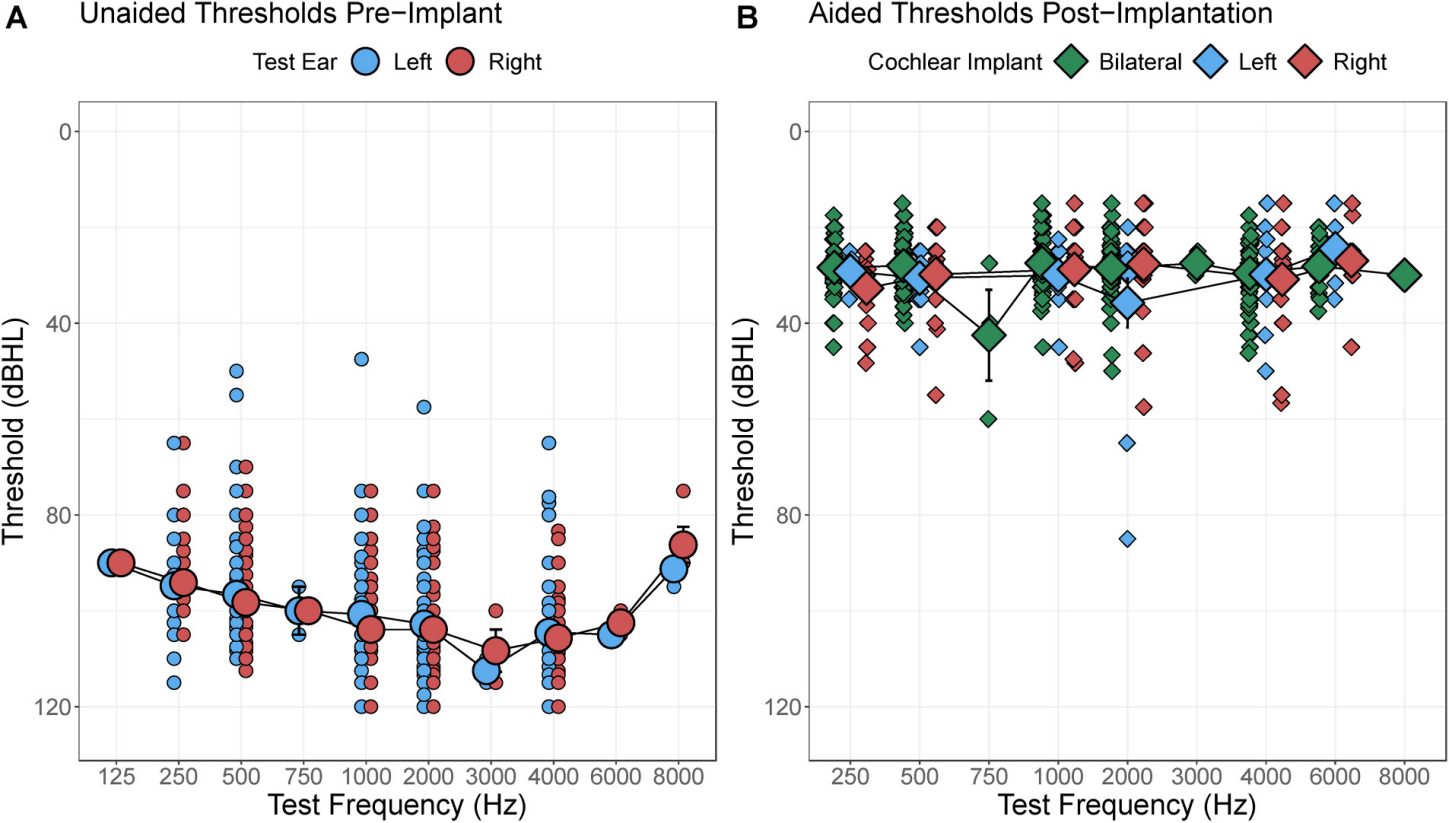
(A) Unaided pure tone thresholds in the ear to be implanted were in severe to profound hearing loss range. (B) Post-operative cochlear implant aided thresholds measured in the soundfield for bilateral, left, or right cochlear implants were in mild hearing loss range indicating good access to conversational speech.

Efficacy of cochlear implantation in infants 9–11 months of age was first assessed by comparing audibility, measured by audiometric pure tone thresholds, before and after cochlear implantation. Figure 2A plots unaided responses from all left and right ears to be implanted (small dots: individual data, large dots: mean and 1SE error bars), revealing severe to profound hearing loss on average. Children with thresholds better than 80 dB HL at some test frequencies were determined to be cochlear implant candidates at their respective implant center site.

Analyses of unaided responses revealed slightly better thresholds from infants at Hearts for Hearing than the other investigational sites (F(4,124.86) = 3.20, p = 0.02) and at low frequencies (F = (8,1066.83) = 31.81, p < 2e-16). Unaided thresholds were not significantly affected by age at audiometric testing (F(1,1111.50) = 3.10, p = 0.08) or ear to be implanted (left vs. right: F(1,1126.23) = 1.51, p = 0.22).

Figure 2B plots aided responses from all left and right ears to be implanted (individual data in small dots, mean and 1SE error bars large dots). Aided thresholds were significantly improved over unaided thresholds (estimate(SE)= 65.04(0.84), t(2946.27) = 77.80, p < 2e-16) with no effect of age at implantation (t(117.53) = 1.32, p = 0.19). Best aided thresholds were found at 1000 Hz (F(5,1728.19) = 4.44, p = 0.0005). There were very small improvements in aided thresholds with duration of implant use (estimate(SE) = −0.88(0.23) dBHL/year, t(1836.48) = −3.76, p = 0.0002) but no significant effects of investigational site (F(4,87.39) = 1.77, p = 0.14) or age at implantation (F(1,87.66) = 0.74, p = 0.39).

**Figure 3.**
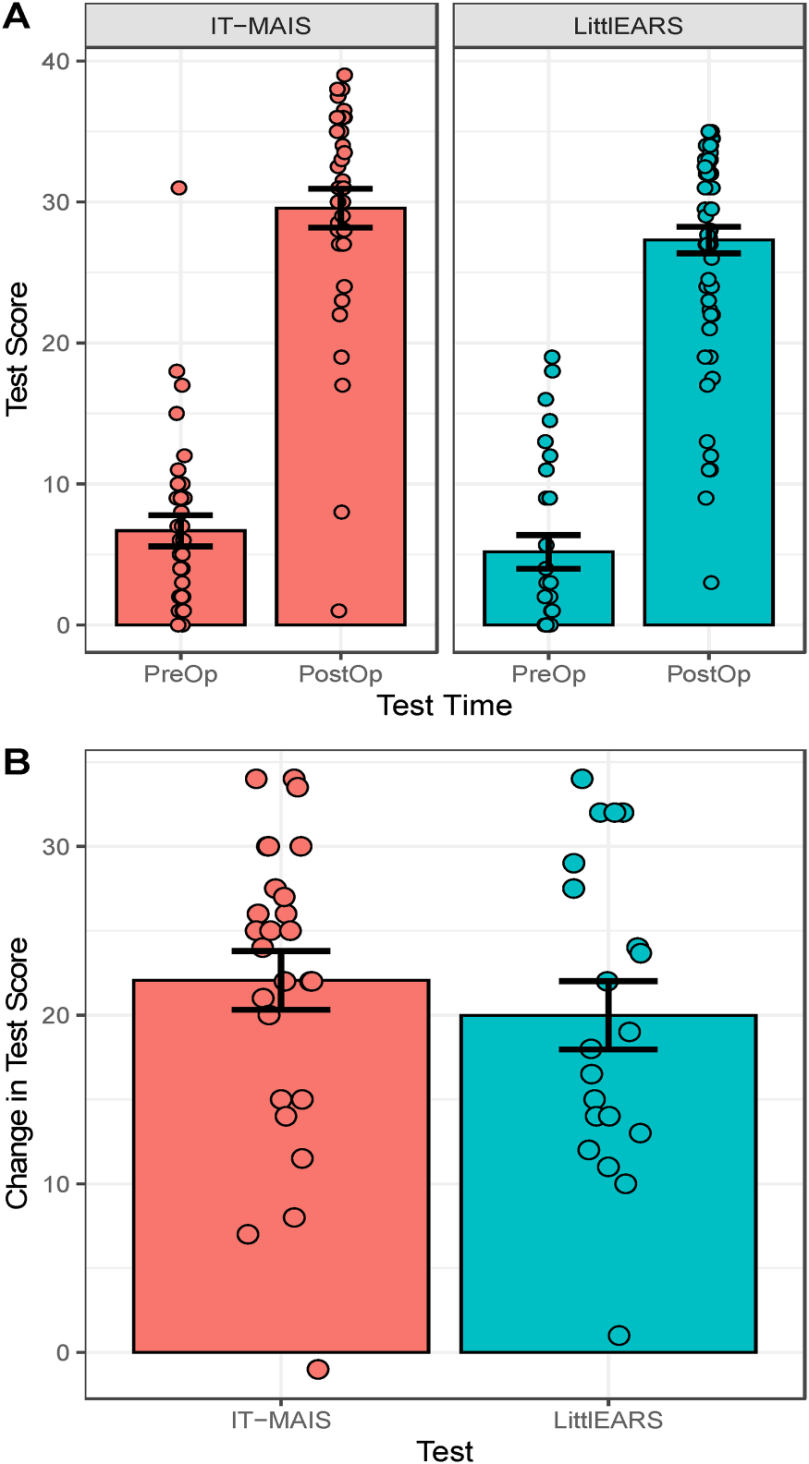
(A) Scores on parent-reported questionnaires of auditory development (IT-MAIS on the left and LittlEARS on the right) were poor pre-implant and improved post-operatively. (B) Change in scores for each child with available data confirmed significant improvement.

Efficacy was also measured by assessing changes in hearing abilities measured by parent-reported questionnaires (IT-MAIS and LittlEARS). Individual (dots) and mean bars with 1SE error bars are shown in Figure 3A for each test at each test time (pre-operative and after cochlear implant use). There is a clear increase from low scores before implantation (Mean(SD) IT-MAIS = 6.7(6.4), Mean(SD) LittlEARS = 5.2(6.3)) to post-implant scores (Mean(SD) IT-MAIS = 29.6(8.3), Mean(SD) LittlEARS = 27.3(7.5)). Differences between test times are shown for each child in Figure 3B. All but one child demonstrated increased scores after cochlear implantation (Mean change (SD) IT-MAIS = 22.1(8.8), Mean change(SD) LittlEARS = 20.0(9.1)). Despite the slight difference in maximum scores possible (35 for LittlEARS and 40 for the IT-MAIS), there was no significant difference in improvement by test (F = 1,17.95) = 1.13, p = 0.30). Test scores significantly increased by estimate(SE)= 17.02(1.11) points/year (t(185.15) = 15.28, p < 2e-16) and this increase was not affected by age at implantation (F1,97.27) = 1.43, p = 0.23); side of unilateral or bilateral implantation (F(2,108.15) = 0.76, p = 0.47); or investigational site (F(4,132.66) = 1.63, p = 0.17).

## Discussion

The present study provided evidence of cochlear implant safety for infants 9–11 months of age over a post-operative period of up to 2 years and demonstrated benefits of cochlear implantation for audibility and early hearing and language development.

Adverse events were reported in 43 (31%) of 143 children studied and totaled 60 events in 227 implanted ears (26%) (Figure 1). Most events were minor (20%) rather than major (6%) and these rates are consistent with previous reports in older children and adults receiving cochlear implants (e.g., Farinetti et al., 2014). The adverse events in the present cohort were managed and resolved but provide some insight into issues that are particular to caring for infants receiving cochlear implants. Temperature regulation during surgery, irritations at the device site, and facial nerve involvements were particular to Cohort 1 and, as reported previously (Deep et al., 2021), largely occurred during the first 6 months following surgery. Lessons from the first cohort were to carefully monitor the temperature of small children during cochlear implant surgery as they had a tendency to be cold as well as to maintain hemostasis, minimize blood loss, and watch for post-operative infection in the form of acute otitis media (Deep et al., 2021). Use of a facial nerve monitor was recommended given the potential to heat the nerve during drilling, resulting in temporary facial nerve injury (Deep et al., 2021).

Ear-related infections occurred in both cohorts (18 events in 14 children), reflecting risks of upper respiratory infections and acute otitis media in this age group (Teele et al., 1989; Vila et al., 2017). Infants who develop an acute otitis media shortly after CI surgery will often present with what appears to be a mastoiditis as a result of the low impedance pathway for infection to spread posterior into the recently drilled mastoid (Osborn et al., 2013). This can be managed with antibiotics with or without tympanostomy tube insertion and at times post auricular incision and drainage for more advanced cases. Such infections do not lead to increase risk of implant loss (Osborn et al., 2013). There were also 19 post-operative events in 15 children and other events such as programming challenges (4 events, 3 children) that were noted in the charts. These were managed by the implant teams and none required hospitalization. This reporting might reflect the careful watch of caregivers of young children after cochlear implantation and a need for the team to provide ongoing care and guidance in the first months and years post-operatively in this young age group. Serious events of CSF leaks (three children) and concerns that required hospital readmission (two children) were not particular to either cohort and occurred at rates previously reported in older children and adults (Loundon et al., 2010; Petersen et al., 2018; Theunisse et al., 2018). As noted in the previous study, CSF leaks are more common in children with cochlear malformations (Deep et al., 2021). Overall, the complications after cochlear implantation in infants 9–11 months were mostly minor and consistent with previous studies examining cochlear implantation in infants younger than 1 year of age (Das Purkayastha et al., 2011; James & Papsin, 2004; Roland et al., 2009).

Efficacy of cochlear implantation in infants was measured by improved audibility and auditory development in parent-reported questionnaires. Figure 2 confirms that infants were provided cochlear implants in ears with severe to profound hearing loss across test frequencies and that cochlear implantation resulted in significant improvements in audibility as measured by aided soundfield thresholds that were largely better than 40 dB HL. Although aided responses measured in this way have been questioned, these measures provide evidence of audibility of speech with hearing aids and cochlear implants (Wiseman & McCreery, 2023; Wiseman et al., 2023). Small improvements in aided thresholds with duration of cochlear implant use and the lack of effect of age at implant could reflect increased auditory sensitivity and/or refinement of the stimulus levels provided in the cochlear implant MAP.

Parent reporting of children's hearing and speech-language behaviors through the IT-MAIS questionnaire and LittlEARS reveal significant improvements over the post-operative period. Mean scores of the IT-MAIS (29.6, 74%) and LittlEARS (27.3, 78%) are consistent with a recent systematic review of speech perception outcomes of cochlear implantation in infants <12 months of age which reported a range of 60–90% for the IT-MAIS and 80–91% for LittlEARS (Wu et al., 2023). The importance here is that these scores are achieved at young ages, thereby reducing gaps from normally hearing peers and improving developmental outcomes at later ages (Cejas et al., n.d.). Indeed, in a recent systematic review of studies in children implanted as infants, Wu et al. reported weighted averages of 79% and 80% for the closed set GASP test and the open set CNC word test (Wu et al., 2023).

Overall, the findings of the present study indicate that complications of cochlear implant surgery in infants are mostly minor and resolve with appropriate management. Surgery in infants does require particular consideration of thermal regulation, blood loss intraoperatively, and risk of infection in the form of acute otitis media postoperatively. The benefits of taking this careful approach are shown to provide clear benefits in audibility and access to spoken language which promotes development of hearing and speech and language. Early implantation is important to reduce gaps relative to normal hearing peers.
